# Glucocorticoid Receptor and Myocyte Enhancer Factor 2 Cooperate to Regulate the Expression of c-JUN in a Neuronal Context

**DOI:** 10.1007/s12031-012-9809-2

**Published:** 2012-05-24

**Authors:** Niels Speksnijder, Kenneth V. Christensen, Michael Didriksen, E. Ronald De Kloet, Nicole A. Datson

**Affiliations:** 1 Division of Medical Pharmacology, Leiden/Amsterdam Center for Drug Research (LACDR), Leiden University Medical Center, P.O. Box 9502, 2300 RA Leiden, The Netherlands; 2 H. Lundbeck A/S R&D, 9 Ottiliavej, DK-2500 Copenhagen-Valby, Denmark; 3 Department of Human Genetics, Leiden University Medical Center, Leiden, The Netherlands

**Keywords:** Glucocorticoid receptor, Myocyte enhancer factor 2, c-JUN, Dexamethasone, PC-12 cells

## Abstract

The glucocorticoid receptor (GR) and myocyte enhancer factor 2 (MEF2) are transcription factors involved in neuronal plasticity. c-JUN, a target gene of GR and MEF2, plays a role in regulating both synaptic strength and synapse number. The aim of this study was to investigate the nature of this dual regulation of c-JUN by GR and MEF2 in a neuronal context. First, we showed that GR mediates the dexamethasone-induced suppression of c-JUN mRNA expression. Next, we observed that GR activation resulted in an increase in phosphorylation of MEF2, a post-translational modification known to change MEF2 from a transcriptional enhancer to a repressor. In addition, we observed an enhanced binding of MEF2 to genomic sites directly upstream of the c-JUN gene upon GR activation. Finally, in primary hippocampal neuronal cultures, knockdown of MEF2 not only reduced c-JUN expression levels but abolished GR regulation of c-JUN expression. This suggests that MEF2 is necessary for GR regulation of c-JUN. In conclusion, for the first time, we show that activated GR requires MEF2 to regulate c-JUN. At the same time, GR influences MEF2 activity and DNA binding. These results give novel insight into the molecular interplay of GR and MEF2 in the control of genes important for neuronal plasticity.

**Electronic supplementary material:**

The online version of this article (doi:10.1007/s12031-012-9809-2) contains supplementary material, which is available to authorized users.

## Introduction

Neuronal plasticity, a change in the structure, function, and organization of neurons in response to environmental stimuli, underlies many key processes such as learning and memory, adaptation, and behavioral sensitization. Changes in gene expression, governed by key transcription factors, such as the glucocorticoid receptor (GR) and myocyte enhancer factor 2 (MEF2), underlie neuroplasticity. GR is activated by glucocorticoid stress hormones, released by the HPA-axis in response to stress. Upon activation, GR acts as a ligand-activated transcription factor to influence expression of a wide variety of genes, including genes involved in neuronal plasticity (Datson et al. 2008). MEF2 comprises a family of four members, MEF2a–d, showing distinct but partly overlapping expression patterns and is activated by neuronal activity. Upon activation, MEF2 regulates the expression of genes that control dendritic remodeling, resulting in the inhibition of synapse formation. Conversely, a decrease in MEF2 activity increases spine density (Flavell et al. 2006; Shalizi et al. 2006).

We previously showed that GR and MEF2 have several target genes in common, including the c-JUN gene (Datson et al. 2011a). c-JUN is a subunit of the transcription factor AP-1 and is a ubiquitously expressed IEG with important functions in cell death, differentiation, and inflammation (Beck et al. 2009; Sun et al. 2005). The AP-1 family of transcription factors is recruited in the activation of neuronal circuits leading to long-term changes, such as long-term memory formation (Alberini 2009). MEF2 is known to induce transcription of c-JUN (Kato et al. 1997; Aude-Garcia et al. 2010; Han and Prywes 1995), while GR on the other hand is known to repress the expression of c-JUN in vitro in AtT-20 cells and mouse fibroblast cells (Autelitano 1994; Wei et al. 1998). The aim of this study was to investigate the molecular interplay of GR and MEF2 in a neuronal context, using the shared target gene c-JUN as a proof-of-principle.

## Materials and Methods

### Cell Culture and Treatment

Rat pheochromocytoma (PC-12) cells (passage # 15–29) were cultured as described earlier (Morsink et al. 2006b). In short, cells were grown in DMEM medium, supplemented with 0–10% fetal bovine serum and 0–10% horse serum, dependent on the stage of neuronal differentiation. For mRNA and protein analysis cells were seeded at a confluency of 30–50% in pre-coated six-well plates (356400, BD Biosciences, San Jose, CA, USA). For ChIP experiments, the cells were seeded at 50% confluency in pre-coated 175 cm^2^ plates (356478, BD Biosciences). Neuronal differentiation was achieved by giving 50 ng/ml Nerve Growth Factor Beta (NGF-ß) (N2513, Sigma-Aldrich, St. Louis, MO, USA) every other day for 10 days. Medium at day 9 of the differentiation was supplemented with charcoal-stripped serum to deprive the medium of endogenous steroids (Sarabdjitsingh et al. 2010b). At day 10, the cells were treated for 30, 60, 90, or 180 min, dependent on the experiment, with either vehicle (VEH) (0.1% ethanol) or 100 nM dexamethasone (DEX) (D1756, Sigma-Aldrich). For GR blockade, cells were pretreated with VEH (0.1% ethanol) or 1 mM RU486 (M8046, Sigma-Aldrich) for 60 min before addition of DEX or VEH.

### Hippocampal Cultures

Newborn pups from NMRI mice were killed at postnatal day 1. Brains were isolated and kept in Hank’s balanced salt solution on ice until dissection. Hippocampi were dissected in ice-cold dissection solution consisting of Krebs buffer supplemented with 3 mg/ml BSA, 1.2 mM MgSO_4_, and 2 mM HEPES. Hippocampi (*n* = 12) were transferred to a conical tube containing 1.5 ml of dissection solution supplemented with 184 μg/ml trypsin. The tissue was incubated at 37°C for 6 min. Subsequently, 3.5 ml of dissection solution supplemented with 0.65 mg/ml soyabean trypsin inhibitor; 10 μg/ml DNAse and 0.19 mM MgSO_4_ were added. The trypsinated and DNase-treated hippocampi were centrifuged at 100×*g* for 3 min. The supernatant was discarded, and the conical part of the tube was filled with 1.5 ml of dissection solution supplemented with 5.2 mg/ml soyabean trypsin inhibitor, 80 μg/ml DNAse, and 1.5 mM MgSO_4_. The cells were dissociated by pipetting and left for 5 min at room temperature (RT), allowing remaining tissue to settle. The supernatant was transferred to a new tube containing 3.5 ml of dissection solution supplemented with 132 μM CaCl_2_ and 120 μM MgSO_4_ and centrifuged for 10 min at 100×*g*. The cell pellet was resuspended in 3.5 ml of MEM II + B27 (MEM buffer supplemented with 0.5% d-glucose, 0.22% bicarbonate_,_ penicillin–streptomycin, 2 mM  l-glutamate, 10% NU-serum, and 2% B27). After resuspension, the concentrated cell solution was diluted to 7.5 ml MEM II + B27. Cells were plated at a density of 50,000 live cells/well in poly-d-lysine coated 96-well dishes. The yield from one pup (two hippocampi) was approximately 400,000 living cells. The day after plating, media was changed to MEM II + B27 buffer supplemented with 1 μM AraC (cytosine arabinoside). The cells were left for 14 days in vitro before assaying.

### Lentiviral shRNA Transduction, Stimulation, and RNA Purification

High titer batches (>5 × 10^−8^ TU/ml) of lentiviral particles harboring gene-specific short hairpin RNA (shRNA) targeting MEF2A (Sigma, TRCN0000095959) as well lentiviral particles harboring control shRNA (Sigma, SHC002V) were purchased from Sigma. The day after plating, hippocampal cultures were transduced with lentiviral particles at the following concentrations: 150,000 lentiviral particles/well, 75,000 lentiviral particles/well, and 37,500 lentiviral particles/well. At day 14, the hippocampal cultures were stimulated for 90 min with 100 nM dexamethasone diluted in astrocyte-conditioned media. The latter was to avoid glutamate-induced excitotoxicity by the media change. Subsequently, cells were processed for RNA isolation using the Aurum Total RNA 96 Kit (BioRad).

### Real-Time Quantitative PCR

Total RNA was isolated using Trizol (15596, Invitrogen) according to the manufacturer’s instructions. RNA was diluted to 50 ng/μl, and cDNA was synthesized using the iScript cDNA synthesis kit (170–8897, Bio-Rad, Hercules, CA, USA) according to the manufacturer’s protocol. Real-time quantitative PCR (RT-qPCR) was performed on a Lightcycler 2.0 real-time PCR system (Roche Applied Science, Basel, Switzerland) in combination with the Lightcycler FastStart DNA Master^PLUS^ SYBR Green I Kit (03515885001, Roche). As a control for genomic contamination, samples without reverse transcriptase were used. The standard curve method was used to quantify the expression differences (Livak and Schmittgen 2001). Expression of TUBB2a (tubulin, beta 2a) was used to normalize the RNA input.

### RT-qPCR Primer Design

Primers were designed using primer-BLAST (NCBI, Bethesda, USA). Apart from the built-in feature of selecting primers that do not cross-hybridize, an additional check for primer hairpins was performed using Oligo 7.0 (MBI Inc. Cascade, USA). Primers were tested with RT-qPCR on a standard curve to check the efficiency of the PCR reaction. After a visual check for single melting peaks, the primer products were put on a 2.0–2.5% agarose gel to check for single products and absence of primer–dimers. The primer sequences used are listed in Table S1.

### Chromatin Immunoprecipitation

The exact procedure is described in Sarabdjitsingh et al. (2010a). In short, cells were fixed with 1% formaldehyde for 10 min at RT to crosslink DNA–protein interactions. DNA was sonicated for 15–25 pulses to obtain DNA fragments between 200 and 500 basepairs and checked visually on a 1.2% agarose gel. Pre-cleared DNA (20–60 μg per antibody) was incubated overnight (o/n) with 6 μg of either anti-MEF2 (sc-313X; Santa Cruz), anti-GR (sc-8992X; Santa Cruz) antibodies, or normal rabbit IgG (Santa Cruz; sc-2027). The next day, 20 μl sepharose A beads were added to the DNA–protein–antibody complexes. The samples were washed 1× with low salt buffer (0.1% sodium dodecyl sulfate (SDS); 2 mM ethylenediaminetetraacetic acid (EDTA) pH 8.0; 20 mM Tris–HCl pH 8.0; 150 mM NaCl; 1% Triton-X-100), 1× with high salt buffer (as low salt except 500 mM NaCl), 1× with LiCL buffer (0.25 M LiCl; 1 mM EDTA pH 8.0; 20 mM Tris–HCl pH 8.0; 1% NP-40; 1% NaDOC), and 2× with TE buffer (1 mM EDTA pH 8.0; 10 mM Tris–HCl pH 8.0). Subsequently, the DNA complexes were eluted from the beads with 0.1 M NaHCO_3_ and 1% SDS, and the DNA was reverse-crosslinked o/n at 4°C in 0.2 M NaCl. The samples were then treated for 1 h with RNAse at 37°C and the DNA purified using Nucleospin columns. The DNA was eluted in TE buffer for RT-qPCR analysis. RT-qPCR on chromatin immunoprecipitation (ChIP) material was performed directly on purified DNA. ChIP results were obtained by performing three individual ChIP replicates. IgG ChIP was used as a negative control for aspecific precipitation while RT-qPCR of myoglobin was used as a negative control for specific precipitation of DNA.

### ChIP Primer Design

Primers were designed spanning a published MEF2 binding site (MBS) upstream of the c-JUN transcription start site (TSS) (Han and Prywes 1995; Haberland et al. 2007). GR binding sites were identified in neuronally differentiated PC-12 cells and rat hippocampus by GR ChIP-sequencing (unpublished data). This resulted in identification of three GR binding sites located ∼300 bp upstream and ∼2 and ∼8 kb downstream the c-JUN TSS (Table 1). All three binding sites were screened for putative glucocorticoid response element (GRE) sequences using an in-house screening method to identify evolutionary conserved GREs (Datson et al. 2011b). Binding of MEF2 and GR to myoglobin was used as a negative control as it is generally considered to be inaccessible for transcription factor binding. Primer sequences used are listed in Table S1.

**Table 1:** ChIP-seq results showing chromosomal locations of the three peaks where increased GR binding was found

Binding site	Chr	Peak start	Peak end	Distance from TSS	GRE sequence	Origin
GBS 1	5	115361507	115361560	−275	None	Hippocampus
GBS 2	5	115359115	115359210	2,097	GAACGGGCTGTGCC	Hippocampus
GBS 3	5	115353332	115353445	7,871	GAACCAAATGTTCA	PC-12 cells

The three sites are designated GBS 1, GBS 2, and GBS 3. ‘Origin’ refers to the ChIP-sequencing experiment in which the GBS was first observed. Distance from TSS refers to the distance between the center of the peak and the transcription start site of c-JUN. A negative value indicates upstream the TSS. The MEF2 binding site (MBS 1) was previously identified (Han and Prywes 1995)

### Western Blotting

Protein was harvested in ice-cold RIPA buffer containing protease inhibitors (#04693124001, Roche) and phosphatase inhibitors (NaVO_3_ and B-glycerophosphate). The cell lysate was incubated on ice for 30 min, spun down, and the supernatant transferred to a new tube. Protein concentration was measured using the Pierce BCA Protein Assay kit (23225, Thermo Scientific, Rockford, IL, USA) according to the manufacturer’s protocol. Diluted samples were supplemented with 1:2 *v/v* of sample buffer (including 2.5% ß-mercaptoethanol and BromoPhenol Blue). Twenty micrograms of each sample was loaded on 10% polyacrylamide gel. After sufficient separation of the proteins, they were transferred o/n at 4°C to a PVDF (polyvinylidene fluoride) membrane. The membrane was subsequently blocked in 5% low fat milk for 1 h at RT for anti-α-tubulin or at or 4°C o/n for phospho-MEF2. Primary antibodies were added in the blocking buffer and incubated for 1 h at RT for anti-α-tubulin or 5 h at 4°C for phospho-MEF2 with either one of the following primary antibodies: anti-phospho S408 MEF2 rabbit monoclonal (ab51151, Abcam, Cambridge, UK), or anti-α-tubulin DM1A mouse monoclonal antibody (T6199, Sigma). Blots were incubated for 1 h at RT with the appropriate secondary antibody: goat-anti rabbit IgG horseradish peroxidase (HRP) secondary antibody (sc-2054, Santa Cruz) or goat-anti mouse IgG HRP secondary antibody (sc-2055, Santa Cruz). Signals were quantified using ImageJ (v1.42; National Institute of Health, USA). α-Tubulin protein expression was used as input normalization.

### Statistics

Statistical analysis was performed with Sigmaplot 11.0 using independent *t* tests in the gene expression studies with/without RU486 pre-treatment. A two-way ANOVA was used with Tukey’s post hoc *t* tests.

## Results

### MEF2a Is Highly Expressed in PC-12 Cells

**Fig. 1:**
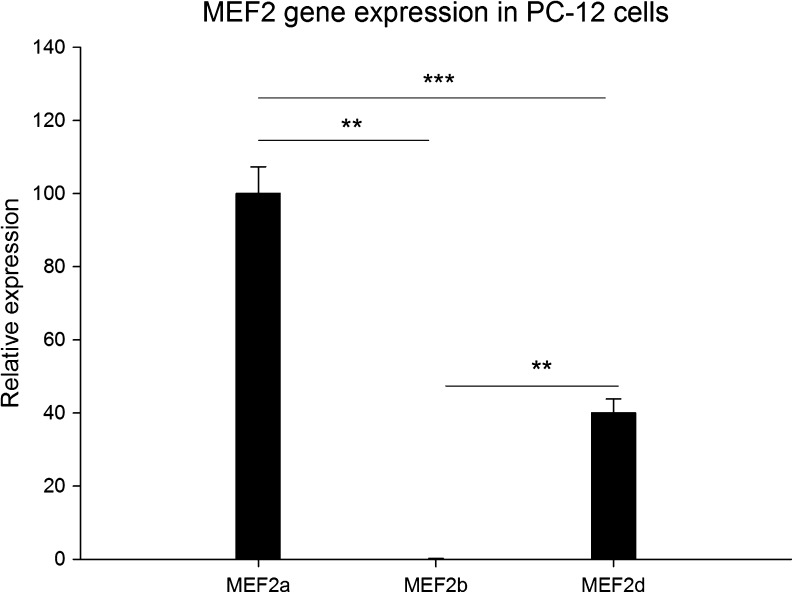
Relative expression levels of transcripts MEF2a, MEF2b, and MEF2d in neuronally differentiated PC-12 cells under VEH conditions (*n* = 6 per group). Expression is relative to MEF2a, which is set at 100%. Expression is normalized to TUBB2a (**p < 0.01; ***p < 0.001 sign. between transcripts)

As a first step to study GR and MEF2 interaction, the endogenous expression of MEF2 transcripts was determined in neuronally differentiated PC-12 cells. MEF2a was most abundantly expressed followed by MEF2d (Fig. 1). MEF2b had a very low expression while MEF2c was not reliably detected in PC-12 cells. Since MEF2a is most ubiquitous, the following experiments focused on this gene.

### GR Activation by DEX Downregulates c-JUN Expression

**Fig. 2:**
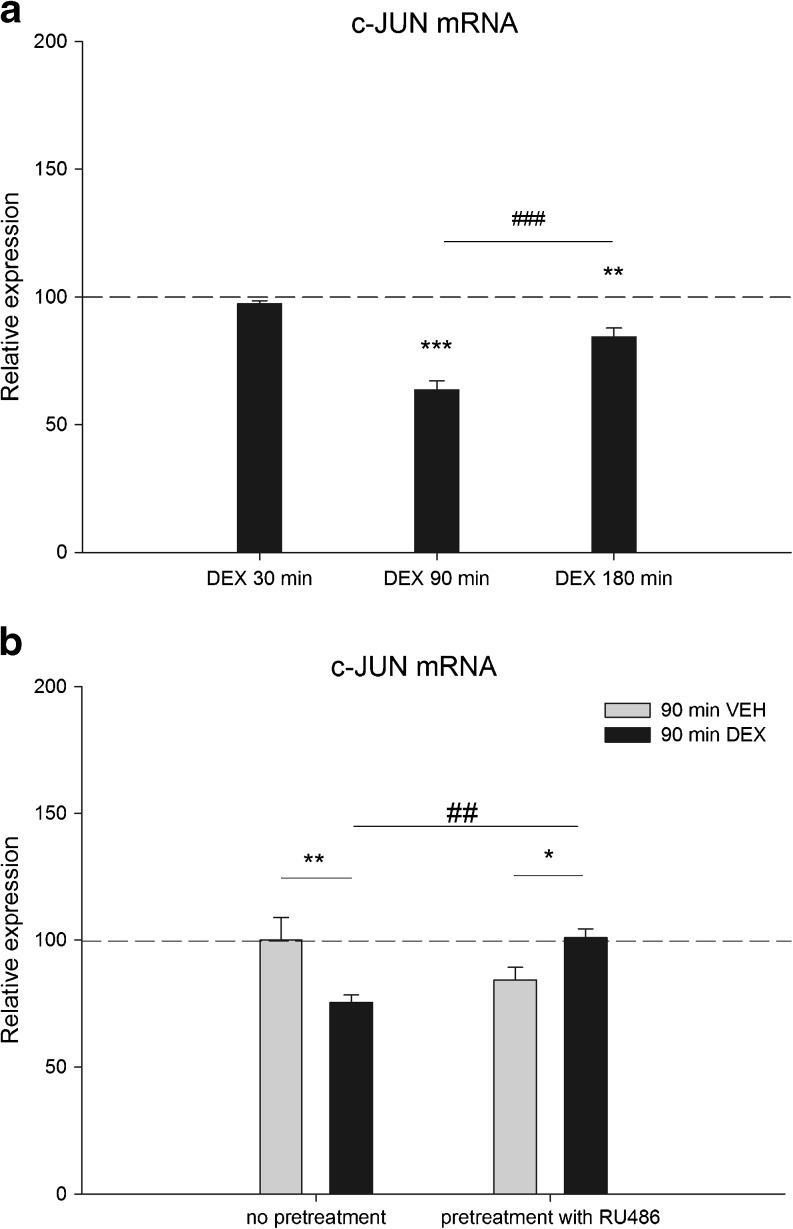
**a** c-JUN mRNA expression at 30, 90, or 180 min of DEX treatment (*n* = 6 per group). For each time point, expression level is relative to its VEH counterpart which is set at 100% and indicated by the *dashed line*. Expression is normalized to TUBB2a (**p < 0.01; ***p < 0.001 sign. vs corresponding VEH treatment) (###*p* < 0.001 sign. between time points) **b** c-JUN mRNA expression at 90 min of DEX treatment with and without 60 min RU486 pretreatment (*n* = 6 per group). Expression level is relative to VEH treated cells without pretreatment, set at 100%. Expression is normalized to TUBB2a (*p < 0.05; **p < 0.01 sign. vs VEH treatment) (##*p* < 0.01 sign. vs DEX without pre-treatment)

Previous studies showed that GR is highly expressed in neuronally differentiated PC-12 cells (Morsink et al. 2006a). To study the GR regulation of c-JUN in a neuronal context, neuronally differentiated PC-12 cells were treated for several time-points with 100 nM of the synthetic glucocorticoid DEX. Expression of c-JUN mRNA was significantly downregulated after 90 min DEX treatment (36% *p* < 0.001). After 180 min, c-JUN expression was significantly higher compared with 90 min DEX (33% *p* < 0.001) but still significantly downregulated (16% *p* < 0.01) compared with the VEH control (Fig. 2a).

### c-JUN Downregulation by DEX Is Mediated by GR

To check whether the DEX effect on c-JUN expression is mediated via GR, PC-12 cells were pretreated with the GR antagonist mifepristone (RU486). Since 90 min DEX treatment showed the largest decrease in c-JUN mRNA expression, PC-12 cells were treated for this period with 100 nM DEX, after being pretreated for 60 min with 1 mM RU486. Again, DEX treatment resulted in a significant downregulation (25% *p* < 0.01) of c-JUN mRNA expression. However, pretreatment with RU486, having no significant effect on its own, completely prevented this effect, showing that the DEX-induced downregulation is mediated via GR (Fig. 2b).

### MEF2a Expression Is Not Changed by GR

**Fig. 3:**
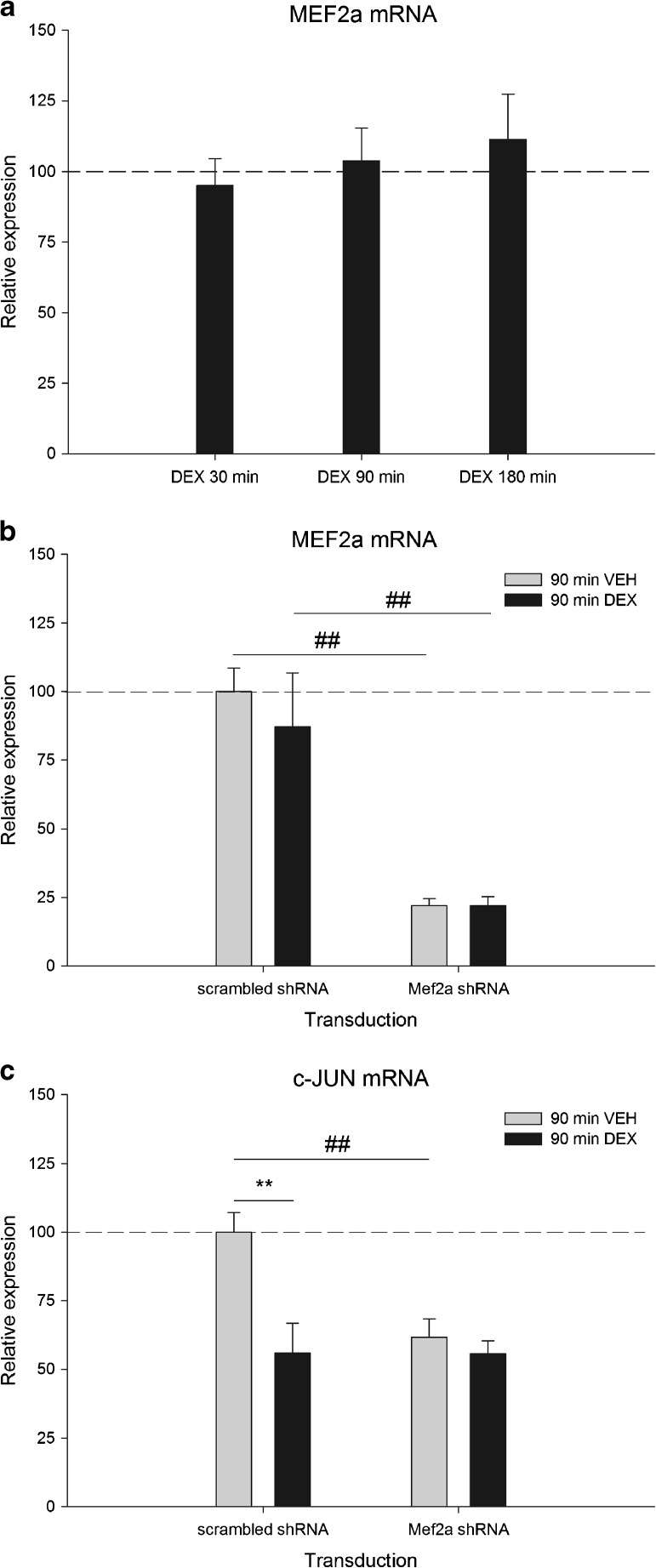
**a** MEF2a mRNA expression in neuronal PC-12 cells at 30, 90, or 180 min of DEX treatment (*n* = 6 per group). For each time point, expression level is relative to its VEH counterpart which is set at 100% and indicated by the *dashed line*. Expression is normalized to TUBB2a. **b** MEF2a mRNA expression at 90 min of DEX treatment in lentiviral transduced primary hippocampal neurons expressing either scrambled shRNA or MEF2a shRNA (*n* = 3 per group). Expression level is relative to scrambled shRNA transduced and VEH treated cells, set at 100%. Expression is normalized to TUBB2a (##*p* < 0.01 sign. vs scrambled shRNA) **c** c-JUN mRNA expression at 90 min of DEX treatment in lentiviral transduced primary hippocampal neurons expressing either scrambled shRNA or MEF2a shRNA (*n* = 3 per group). Expression level is relative to scrambled shRNA transduced and VEH-treated cells, set at 100%. Expression is normalized to TUBB2a (**p < 0.01 sign. vs VEH treatment) (##*p* < 0.01 sign. vs scrambled shRNA)

Since c-JUN is also a known MEF2 target gene, we tested whether DEX treatment changed the expression of MEF2a in PC-12 cells. MEF2a expression showed no change following DEX treatment at the time points studied (Fig. 3a).

### MEF2a Is Necessary for the GR-Mediated Effect on c-JUN

To examine whether MEF2a is necessary for the DEX effect on c-JUN expression, we aimed to knock down MEF2a in PC-12 cells before treatment with DEX. Although MEF2a could be knocked down in non-differentiated PC-12 cells, it failed when cells have a neuronal phenotype (data not shown). Since MEF2 proteins are involved in regulation of the neuronal phenotype (Shalizi et al. 2006; Lin et al. 1996; Tian et al. 2010), as well as in neuronal viability (McKinsey et al. 2002), we did not consider knocking down of MEF2a before differentiation to be a good alternative. Instead, the involvement of MEF2a in DEX-mediated effects on c-JUN gene expression was evaluated in primary hippocampal cultures using lentiviral shRNA-mediated MEF2a knockdown. Hippocampal cultures were transduced and incubated with lentiviral particles harboring either negative control shRNA (scrambled sequence) or a gene-specific shRNA targeting MEF2a followed by a 90 min 100 nM DEX treatment. Gene expression measurements revealed a significant knockdown of MEF2a (78% *p* < 0.001) in VEH-treated cells compared with cells transduced with negative control shRNA (Fig. 3b). DEX treatment did not influence MEF2a expression, neither in the control condition nor in MEF2a shRNA transduced cells. c-JUN expression showed a significant downregulation after DEX in control cells (44% *p* < 0.01) (Fig. 3c), in accordance with our findings in PC-12 cells (Fig 2b). Knockdown of MEF2a, however, resulted in a downregulation of c-JUN comparable to the effect of DEX in control cells (38% *p* < 0.001). Surprisingly, DEX treatment on top of knocked down MEF2a did not result in any additional knockdown.

### MEF2a Phosphorylation Is Increased by GR

**Fig. 4:**
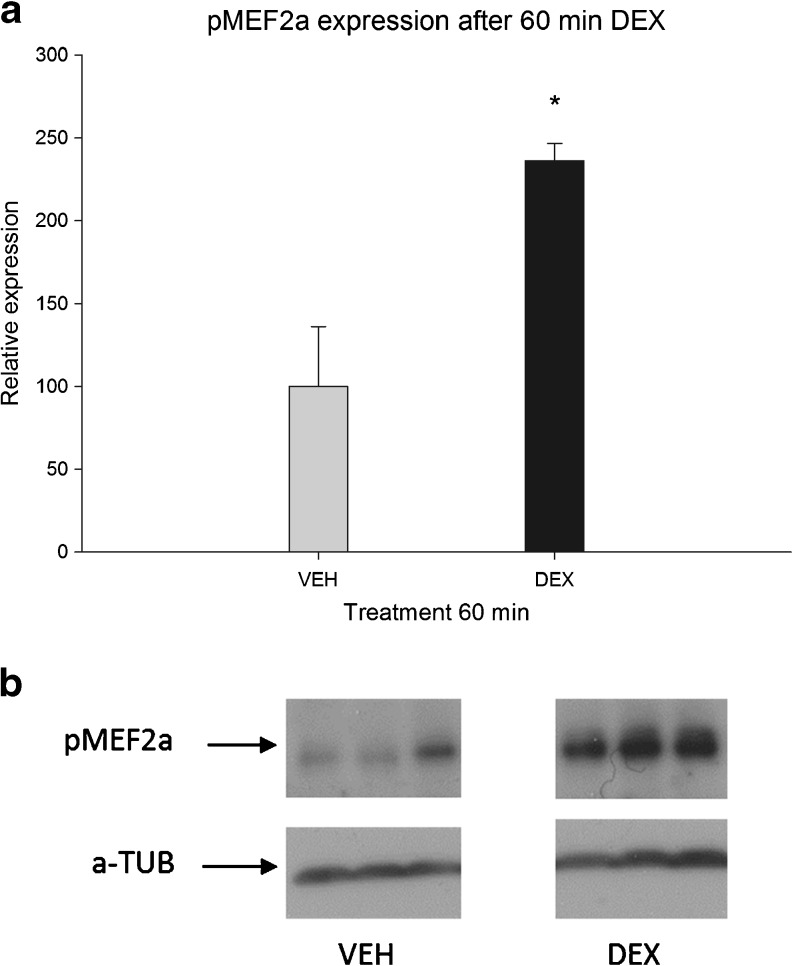
MEF2a S408 phosphorylation level after 60 min of DEX treatment (*n* = 3 per group). **a** Phosphorylation level is relative to 60 min VEH treatment, set at 100%. Expression is normalized to alpha-tubulin (*p < 0.05 sign. vs VEH treatment). **b** Representative example of protein expression

Many studies have shown the importance of phosphorylation of serine 408 in MEF2a for the activity of MEF2 (Shalizi et al. 2006; Gregoire et al. 2006; Li et al. 2001). An increased ratio of phosphorylated versus dephosphorylated MEF2a has been shown to decrease MEF2 transcriptional activity. Therefore, we measured this important hallmark after 60 min of 100 nM DEX treatment (Fig. 4a, b) in neuronally differentiated PC-12 cells. DEX treatment induced a marked increase in phosphorylation of MEF2a compared with VEH-treated cells (125% *p* < 0.05). An independent experiment showed comparable changes in phosphorylation while, at 180 min, no difference in phosphorylation was detected anymore (results not shown).

### GR- and MEF2-DNA Binding Are Increased Around the c-JUN Gene

**Fig. 5:**
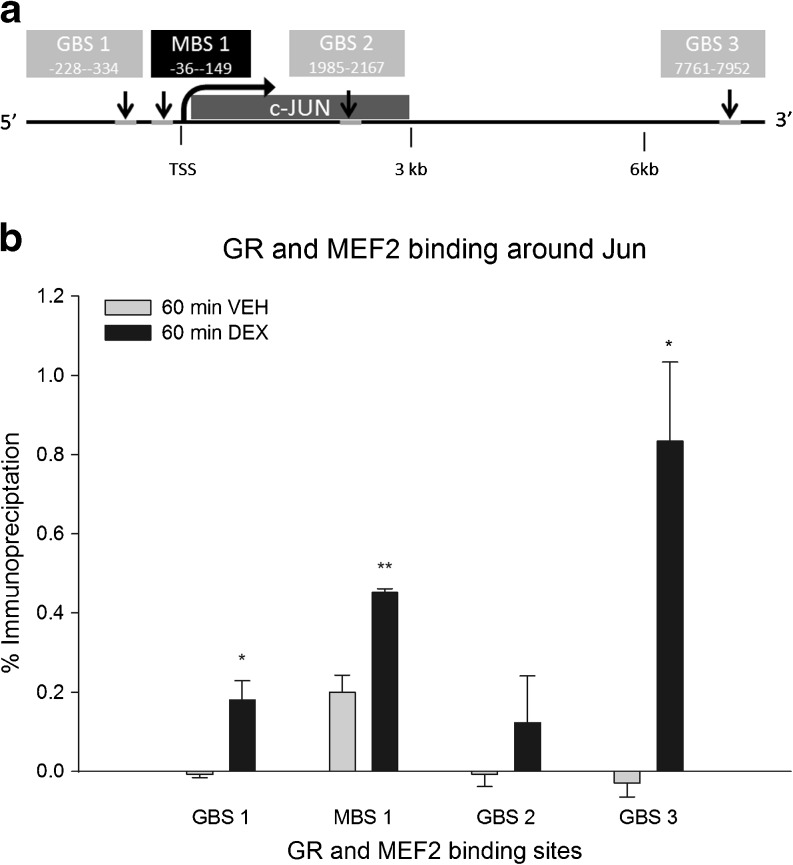
Binding levels of GR and MEF2a in the vicinity of the c-JUN gene (*n* = 3 per group). **a** Schematic overview of the c-JUN gene and surrounding sites. GR binding sites 1, 2, and 3 are depicted in *grey* and the MBS1 is depicted in *black*. *Numbers inside* the *boxes* indicate distance from the beginning and end of the peak to the TSS. **b** ChIP results representing DNA-binding of GR at three distinct binding sites designated GBS1, GBS2, and GBS3, and DNA-binding of MEF2a at the MEF2-binding site designated MBS1. Results are immunoprecipitated fractions plotted as percentage of total input DNA. The immunoprecipitated fraction is normalized to IgG binding (*p < 0.05, **p < 0.01 sign. vs VEH treatment)

MEF2 and GR are well-known transcription factors and exert their action by binding directly or indirectly to the DNA. First, we investigated whether GR activation changed the binding of MEF2 to a previously described MEF2 binding site (MBS) upstream of c-JUN (Han et al. 1992) using ChIP. In addition, we identified three novel GR binding sites (GBS) based on ChIP-Seq data for GR in PC-12 cells as well as rat hippocampus (unpublished data): a GBS 300 bp upstream to c-JUN (GBS1) and located within a short distance (< 100 bp) from the MBS and another two GBS ∼2 and ∼8 kb downstream of the c-JUN TSS (GBS2 and GBS3, respectively) (Table 1 and Fig. 5a). We investigated whether activated GR showed binding to these sites. To this end, neuronally differentiated PC-12 cells were treated for 60 min with 100 nM DEX, and DNA–protein complexes were immunoprecipitated using GR or MEF2 antibodies. MEF2 binding to the upstream MBS was increased after 60 min DEX treatment (2.26-fold *p* < 0.01) (Fig. 5b). Moreover, DEX treatment increased GR binding to both GBS1 and GBS3 (*p* < 0.05 for both GBS1 and GBS3) but not to GBS2 (Fig. 5b). Binding of GR and MEF2 to a control region (myoglobin) was not enhanced after DEX treatment (results not shown). Screening of the GBS for putative GREs revealed presence of a GRE at the downstream GBS 3, but not in GBS1.

## Discussion

GR and MEF2 are both transcription factors known to influence neuronal plasticity. We previously observed that GR and MEF2 have several target genes in common, including c-JUN, and hypothesized that both transcription factors may cooperate in a neuronal context in the regulation of genes important for plasticity. Here, we present evidence that there is an interplay of GR and MEF2 in the regulation of c-JUN at multiple levels. Our results show that activation of GR regulates phosphorylation and hence, transcriptional activity of MEF2a as well as MEF2a-DNA binding upstream of target gene c-JUN.

### In Vitro Model

To study GR and MEF2 effects on target gene c-JUN, we used neuronally differentiated PC-12 cells, a frequently used neuronal cell model. Previous studies showed that both GR and MEF2d are highly expressed in this cell line (Morsink et al. 2006a; Kim et al. 2011). Here we show here that the MEF2a isoform, which is highly expressed in the limbic system, has even higher expression levels than MEF2d. Therefore, we considered neuronal PC-12 cells to be a good model system to study the interaction of MEF2 and GR signaling in a neuronal context. Since lentiviral- or siRNA-mediated knockdown of MEF2a proved to be difficult in this cell line after differentiation to a neuronal phenotype, we decided to use primary hippocampal neurons to study the effect of MEF2a knockdown on c-JUN expression. Note that DEX downregulates c-JUN to the same extent in both cell lines.

### c-JUN mRNA Regulation as Proof-of-Principle for MEF2 and GR Interplay

To study the effect of GR on MEF2 activity and DNA-binding, we focused on the AP-1 transcription factor subunit c-JUN for several reasons. AP-1 is a ubiquitously expressed transcription factor and an important mediator of activity-induced dendritic growth (Hartwig et al. 2008). MEF2 is also a mediator of dendritic growth (possibly via c-JUN) and enhances the expression of c-JUN in an activity-dependent manner (Flavell et al. 2008). Indeed, we show that acute activation of GR by DEX downregulates the expression of c-JUN, which is possibly mediated by decreased transcriptional activity of MEF2a due to phosphorylation.

### MEF2a Is Necessary for the GR-Mediated Effect on c-JUN Transcription

Knockdown of MEF2a led to a decrease in expression of c-JUN, implying that expression of c-JUN is mediated by MEF2a under vehicle conditions. This idea is strengthened by our ChIP results which indicate that MEF2 is already bound to the MBS under VEH conditions. Other transcription factors than MEF2a likely also play a role, since the downregulation of c-JUN was relatively small compared with the knockdown of MEF2a. Indeed, MEF2d and MEF2c are also able to regulate c-JUN expression and are therefore possible candidates controlling c-JUN expression. Interestingly, DEX treatment on top of MEF2a knockdown had no additional effect whatsoever, suggesting that the GR cannot exert its effect after knockdown of (phosphorylated) MEF2a.

### Transcriptional Machinery Is Repressed by Phosphorylated and DNA-Bound MEF2a

Several studies have shown that post-translational modification of MEF2 significantly alters its activity (Molkentin et al. 1996; Shalizi et al. 2006; Gregoire et al. 2006). Phosphorylation of serine 408 in MEF2a has an inhibitory effect on MEF2 transcriptional activity (Flavell et al. 2006; Gong et al. 2003; Shalizi et al. 2006; Potthoff and Olson 2007). Conversely, dephosphorylation of serine 408 in MEF2a is induced by neuronal activity, leading to activation of calcineurin, a potent phosphatase of MEF2 (Flavell et al. 2006; Shalizi et al. 2006). We show here for the first time that DEX treatment increases phosphorylation of MEF2a.

The DEX effect on phosphorylation and hence decreased activity of MEF2a and increased DNA binding at the same time, described in this study, seem contradictory at first. However, results on phospoinositide-3-kinase, catalytic, gamma polypeptide (PIK3CG) binding and expression reveal a similar response pattern. Pulipparacharuvil et al. showed that increased phosphorylation of MEF2a at S408 correlated with increased binding of MEF2 close to the TSS of PIK3CG and decreased expression of the transcript (Pulipparacharuvil et al. 2008). In agreement with this, McKinsey et al. showed that activated MEF2 is able to recruit histone acetyl transferases (HATs) such as p300, while phosphorylated MEF2 recruits histone deacetylases (HDACs) such as HDAC 4, 5, 7, and 9. MEF2 thus operates as a switch and is therefore able to directly activate or repress the transcriptional machinery (McKinsey et al. 2002).

**Fig. 6:**
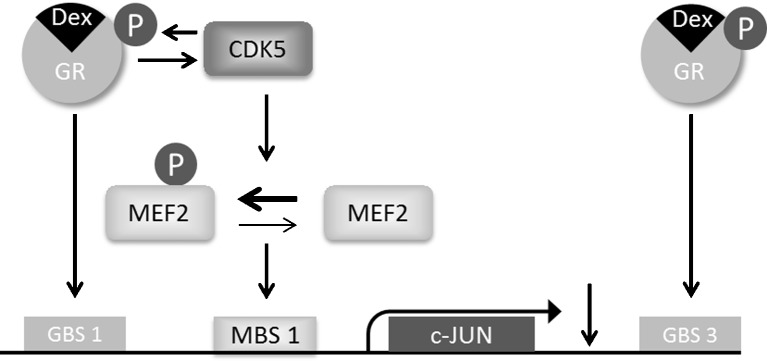
Schematic overview of the obtained results. Activation of GR by agonist DEX activates the kinase CDK5, responsible for phosphorylation of its target proteins, including GR and MEF2. MEF2, now phosphorylated, is increasingly bound to the DNA where it is suggested to act as a transcriptional repressor. GR is also bound to the DNA under this condition. However, to what extent GR itself is responsible for c-JUN downregulation remains to be studied

A similar mechanism may be involved in regulation of c-JUN (Fig. 6). At 60 min of DEX treatment, MEF2a is phosphorylated and bound to the DNA to a higher extent than under VEH conditions. This may imply that the transcriptional machinery is repressed, probably by attracting HDACs. At the same time, DNA binding of GR to GBS1, in the vicinity of MEF2a, is also increased, likely due to indirect binding via an intermediate transcription factor, since this site was not shown to contain a putative GRE. It was recently shown that increased HDAC7-DNA binding within exactly the same region as the GBS1 results in c-JUN downregulation in a deacetylase-independent manner (Ma and D’Mello 2011), suggesting that HDAC attraction may repress the transcriptional machinery without preventing other transcription factors to bind to the DNA.

GC effects on the transcriptional machinery may represent a more general phenomenon underlying some of the long-term changes in neuronal expression that have been observed in response to acute GR activation. For example, in the CA1 area of the hippocampus, long-term potentiation was found to be enhanced up to 24 h after a brief stress-induced rise in corticosterone, accompanied by an enhanced expression of GR that was still present 24 h after termination of the stress response (Ahmed et al. 2006). Another study, focusing directly on the plasticity-related gene neuropsin, found that corticosterone readily upregulates this gene in the hippocampus, which remains elevated for over 24 h (Harada et al. 2008). Even weeks after stress, a persistent overexpression of the stress-associated splice variant of the neuronal acetylcholinesterase gene was observed, likely caused by long-term expression of the SC35 splicing factor in response to stress (Meshorer et al. 2005).

Exactly how activated GR leads to increased phosphorylation of MEF2a is not known. The mainly neuron-specific kinase CDK5 phosphorylates MEF2a at serine 408 (Gong et al. 2003). Since GR is known to recruit CDK5 for its own phosphorylation upon DEX binding (Kino et al. 2007), we hypothesize that MEF2a is recruited at the same time by GR and hence is phosphorylated by CDK5. Alternatively, CDK5 activity may be enhanced upon binding to GR and subsequently, after detaching from GR, starts to phosphorylate other target proteins like MEF2a. Furthermore, it has been shown that calcineurin (CaN) mRNA expression, a phosphatase responsible for reducing phosphorylation at serine 408 in MEF2a, is significantly reduced by corticosterone treatment (Morsink et al. 2006b), which might also lead to increased phosphorylation levels. However, downregulation of CaN was only observed at 180 min of corticosterone treatment, while the present study indicates a phosphorylation difference already at 60 min of DEX treatment.

Another possibility of decreased transcriptional activity by MEF2 might be the downregulation of transcriptional enhancers like the previously mentioned p300. This HAT is a direct target of microRNA-132 which was found to be extensively upregulated under stressful conditions (Shaltiel et al. 2012). MEF2 plays an important role in neuronal differentiation (Shalizi and Bonni 2005). Since miR-132 was also recently found to play an important role in neuronal differentiation of PC-12 cells as well as of hippocampal neurons, it is possible that elevated corticosterone levels influence MEF2 function via this pathway as well (Luikart et al. 2011; Magill et al. 2010).

## Conclusion

This study provides new insights into the molecular interplay at multiple levels of two transcription factors that are central to neuronal plasticity, GR and MEF2a. To our knowledge, this is the first report showing a direct effect of GR on the activity and DNA-binding of MEF2a. An interesting avenue for future studies will be to determine how stress and subsequent glucocorticoid release influences MEF2 in several brain areas and how this might affect plasticity-based processes such as learning and memory.

## Electronic supplementary material

Below is the link to the electronic supplementary material.

Table S1 Primer sequences used for gene expression measurements or immuno-precipitated DNA fragments bound by GR or MEF2 (XLSX 12 kb)

## Abbreviations

CDK5: Cyclin-dependent kinase 5
ChIP: Chromatin immuno-precipitation
DEX: Dexamethasone
GBS: GR-binding site
GR: Glucocorticoid receptor
GRE: Glucocorticoid response element
HPA-axis: Hypothalamic–pituitary–adrenal axis
IEG: Immediate-early gene
MBS: MEF2 binding site
MEF2: Myocyte enhancer factor 2
PC-12: Pheochromocytoma cells
